# The Role of Nitroreductases in Resistance to Nitroimidazoles

**DOI:** 10.3390/biology10050388

**Published:** 2021-05-01

**Authors:** Carol Thomas, Christopher D. Gwenin

**Affiliations:** 1 School of Natural Sciences, Bangor University, Bangor LL57 2UW, UK; carol.thomas@bangor.ac.uk; 2 Department of Chemistry, Xi’an Jiaotong-Liverpool University, 111 Ren’ai Road, Suzhou Industrial Park, Suzhou 215123, China

**Keywords:** nitroreductases, antimicrobial resistance, mitromidazole, metronidazole, nim genes

## Abstract

**Simple Summary:**

Antimicrobial resistance continues to be a major global health threat. It is estimated by the WHO that 700,000 people die each year because of drug resistance, and this is predicted to rise to 10 million by 2050. As well as the increased cost, which is forecast to exceed $100 trillion, as more expensive drugs have to be deployed, illnesses often last longer and require hospital treatment. This, in turn, increases the strain on often-inadequate healthcare systems. As resistances continue to grow, finding alternatives is crucial. This review showed that nitroreductases play a role in drug activation but are also associated with resistance mechanisms. These mechanisms require further investigation to fully understand them before they can be utilised against multidrug-resistant organisms. This will depend on committed collaborations between the private and public sector to translate academic research into the clinic.

**Abstract:**

Antimicrobial resistance is a major challenge facing modern medicine, with an estimated 700,000 people dying annually and a global cost in excess of $100 trillion. This has led to an increased need to develop new, effective treatments. This review focuses on nitroimidazoles, which have seen a resurgence in interest due to their broad spectrum of activity against anaerobic Gram-negative and Gram-positive bacteria. The role of nitroreductases is to activate the antimicrobial by reducing the nitro group. A decrease in the activity of nitroreductases is associated with resistance. This review will discuss the resistance mechanisms of different disease organisms, including *Mycobacterium tuberculosis*, *Helicobacter pylori* and *Staphylococcus aureus*, and how these impact the effectiveness of specific nitroimidazoles. Perspectives in the field of nitroimidazole drug development are also summarised.

## 1. Introduction

Nitroreductases (NTR) are a family of proteins involved in the reduction of nitro-containing compounds [1]. The flexibility of these enzymes comprises their usefulness in a variety of biological and medical applications. These include environmental decontamination using bioremediation [2]; various cancer therapies such as gene and viral-directed prodrugs [3,4,5,6,7] and probes for detecting hypoxia in tumours [8,9,10,11], antiparasitics [12,13], herbicides [14] and for the detection of explosives [15]. For a more detailed review of the many uses of nitroreductases, see the papers by Kumari et al. [11], Zhang et al. [4] and Nepali et al. [16].

**Figure 1:**
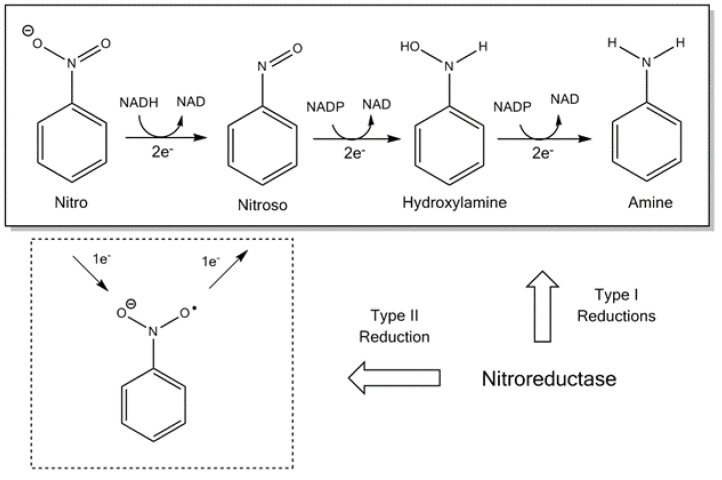
The reduction scheme of a nitro group and the respective electron transfers required [20]. NAD: nicotinamide adenine dinucleotide and NADPH: nicotinamide adenine dinucleotide phosphate.

Nitroreductases can be divided into two groups: flavin reductases [17] and those from enteric bacteria [18]. Typical NTRs share similar biochemical properties; they usually occur as a homodimer, contain flavin mononucleotide (FMN) as a cofactor and catalyse using the ping-pong bi-bi kinetic mechanism [1,19]. The bacterial NTRs can be further split into Type I, oxygen-insensitive and type II, which are oxygen-sensitive, as illustrated in Figure 1 [20]. Bacteria can contain both type I and II, although the most studied are those enzymes that belong to type I [19]. The oxygen-insensitive NTRs can be further divided into major and minor protein groups. The minor group can utilise nicotinamide adenine dinucleotide (NAPH) or nicotinamide adenine dinucleotide phosphate (NADPH), while the major group can utilise NADPH only as the electron donor [21]. The most studied examples of the major and minor groups are the *Escherichia coli* NfsA and NfsB enzymes [21].

The physiological functions of NTRs are not completely understood, but several have been proposed for oxygen-insensitive bacterial NTRs [19]. It has been assumed that they have a role to play in detoxification, as they reduce a broad range of compounds [19]. Some are involved in specific degradation pathways, such as nitrobenzene reductase and nitrophenol reductase [22,23]. NTRs may also be involved in the response to oxidative stress, as the enzyme NfsA is regulated by the SoxRS system, which is involved in the prevention of oxidative damage [24,25]. The range and adaptability of NTRs have enabled some of these enzymes to specialise in different metabolic functions without necessarily losing the other reductase activities [19]. Therefore, while some NTRs are associated with specific metabolic pathways or the reduction of different nitroaromatic compounds, others may be active in processes such as oxidative stress response or bioluminescence [26]. Due to the ability of NTRs to affect the toxic, mutagenic and carcinogenic characteristics of many nitroaromatics, nitrofuran derivatives have been used to develop a group of antimicrobials commonly knowns as nitroimidazoles [27].

Nitroimidazoles have seen a resurgence in interest due to their broad spectrum of activity against anaerobic Gram-negative and Gram-positive bacteria. They are particularly being seen as important in the battle against antibiotic resistance. Resistance to antibiotics is a major challenge facing modern medicine, and its importance was highlighted in 2016 when it was a topic for discussion at the United Nations General Assembly [28]. It is estimated that 700,000 people die each year because of infections that are resistant to current antibiotics and that this figure is predicted to rise to 10 million annually by 2050, with the global cost forecast to exceed $100 trillion over the next few decades [29]. The overuse of antibiotics in both clinical and agricultural settings has accelerated the process of resistance [29,30]. Perhaps the most widely known antibiotic is penicillin, which was discovered by Fleming in 1928, although it was not produced on any great scale until 1940 [30]. Most current antimicrobials were discovered between the 1940s and 1970s, including nitroimidazoles in the early 1950s, when azomycin was isolated from a crude extract of *Streptomyces* bacteria [31]. This review will look at the role played by NTRs in the resistance mechanisms of nitroimidazoles.

## 2. Nitroimidazoles

**Figure 2:**
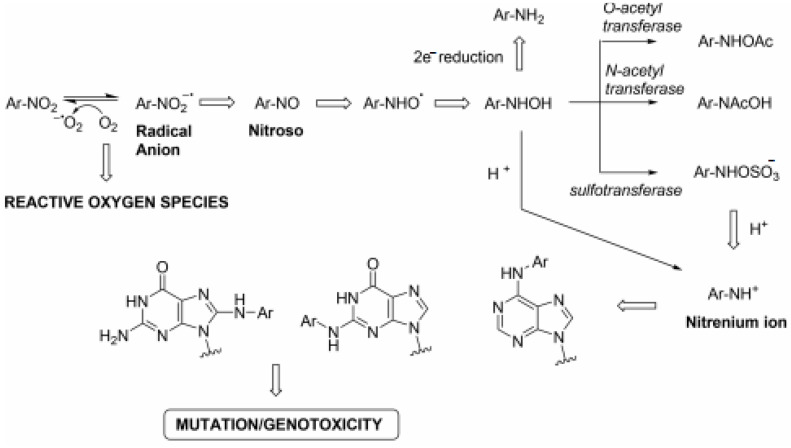
Mutagenic pathway of nitroarenes [33].

Nitroimidazoles are a class of antimicrobial drugs that have a broad spectrum of activity against anaerobic Gram-positive and Gram-negative bacteria, as well as parasites and mycobacteria, since their discovery drugs such as metronidazole, pretomanid and delamanid have gone on to form a large part of the treatment for *Helicobacter pylori* and *Mycobacterium tuberculosis,* respectively. The mode of action of nitroimidazoles can help to explain why they have such a broad spectrum of activity. They are prodrugs that require the reduction of the nitro group before they display any antimicrobial effects. This is normally achieved by NTRs using flavin mononucleotide (FMN) or flavin adenine dinucleotide (FAD) as prosthetic groups and either NADH or NADPH as reducing agents [27]. The mechanism is understood to have the following steps: (i) molecules enter the cells through passive diffusion, (ii) the nitro group is reduced to reactive radical species and (iii) the radicals react with the DNA or protein within the cell [27]. The reduction of products within a cell depends on the redox potential of the compound and the number of electrons being transferred [32]. The system operates more efficiently under anaerobic conditions, meaning the bactericidal effects are increased compared to when oxygen is present [33]. The complete pathway can be seen in Figure 2. The diverse mode of action, which is often inadequately defined, can lead to problems when trying to maximise their bactericidal potential. It is also a factor that must be taken into consideration when looking at the important issue of resistance.

Resistance to nitroimidazoles usually occurs because there is a decrease in the activity of the enzymes responsible for the reduction of the nitro group [34]. These resistance mechanisms differ depending on the target organism and will be described in more depth in the sections below. To develop new drugs that will aid the management of infectious diseases, these resistance mechanisms must be better understood.

## 3. Metronidazole

**Figure 3:**
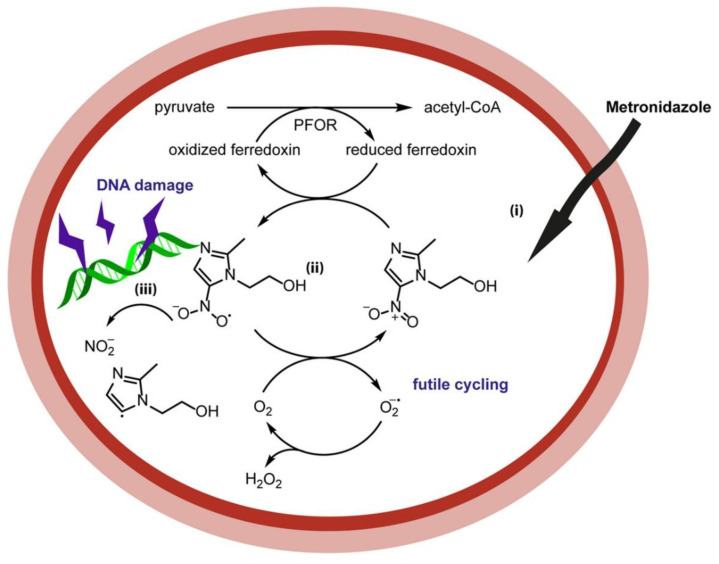
Mechanism of metronidazole [27] involving bioreduction of the nitro group by ferredoxin.

Metronidazole (MTZ) is a nitroimidazole prodrug derived from azomycin, which has been used as an antimicrobial since the early 1960s [35]. The drug enters the cells via passive diffusion but is inactive until the nitro group is reduced. This can occur via two routes (Figure 3): reductive activation, which results in toxicity, or reductive inactivation, where the nitro group is reduced to a nontoxic amino derivative [19,36]. MTZ is one of the main drugs used to treat *Helicobacter pylori*, which is a Gram-negative microaerophile bacterium that can be found in the stomach of almost 50% of the world’s population. The symptoms include chronic gastritis and peptic ulcers. It is also classed as a type I carcinogen by WHO, as it is a risk factor in gastric adenocarcinoma and mucosa-associated lymphoid tissue lymphoma (MALT) [37,38,39].

Resistance to MTZ in cases of *H. pylori* is more common than with other antimicrobials and can be as high as 80%, depending on the patient group and geographic region [40]. As the mechanism of action requires enzymatic reduction, anything which affects this can cause resistance. Mutations in the genes that encode certain electron transport proteins are one such example; inactivation of the *rdxA* (encodes oxygen-insensitive NADPH nitroreductase) and *frxA* (encodes NADPH flavin oxidoreductase) genes is linked to MTZ resistance in *H. pylori* [41,42]. Chua et al. [43] demonstrated a strong correlation between mutations that inactivate RdxA and its resistance. They also reported that a significant number of MTZ-resistant strains of *H. pylori* had a mutation of the Arg-16 residue of RdxA. This amino acid residue is responsible for binding between RdxA and the FMN phosphoryl group, and therefore, any mutation may adversely affect the reduction of MTZ to its cytotoxic form [43]. However, this cannot be responsible on its own for conferring MTZ resistance, as it has also been found in *H. pylori* strains that were susceptible to MTZ [43]. Chua et al. [43] proposed that high levels of *frxA* would counteract the effect of Arg-16 and make *H. pylori* susceptible to MTZ, despite any mutation of *rdxA.* If *frxA* is inactivated, it does not appear to play a major role in MTZ-resistance but may work together with other mutations to increase the resistance [43].

As resistance to MTZ can occur without either *rdxA* or *frxA* inactivation, other mechanisms must be considered [44,45]. One such mechanism suggested by Lee et al. [46] is a gene, *hefA*, associated with the efflux pump HefABC. The expression of this gene is increased when exposed to MTZ. In MTZ-resistant strains of *H. pylori* with intact *frxA* and *rdxA*, the levels of *hefA* were higher than in susceptible strains. If *hefA* was knocked out, the strains became sensitive to MTZ, and this was reversed by the addition of *hefA,* showing that *hefA* was directly involved in MTZ-resistance [46]. While this could help to explain cases of MTZ resistance in the presence of functioning *frxA* and *rdxA* genes, the overexpression of *hefA* alone does not result in resistance [46]. Clearly, the resistance of *H. pylori* to MTZ is a complex mechanism that requires further study to fully understand.

Nitroimidazole resistance at low levels is often associated with the *nim* genes [47]. The *Nim* genes were first described in 1994 [48], and their nitroreductase activity was observed two years later when a *nimA*-positive strain of *Bacteroides fragilis* reduced a nitroimidazole drug to its noncytotoxic amine derivative [49]. Currently, eleven *nim* genes have been identified: *nimA*–*nimK* [50].

**Figure 4:**
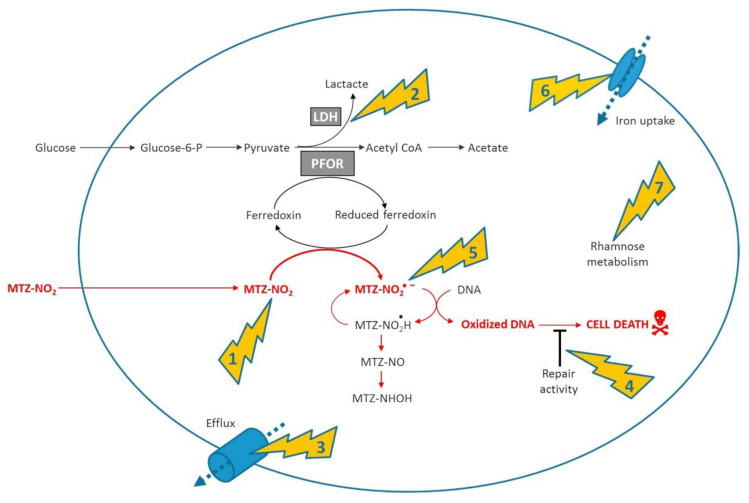
Summarised mode of action and main mechanisms involved in resistance. (**1**) Shows nitroimidazole reductase activity encoded by the *nim* genes, (**2**) metabolic shift away to the pathway related to conversion of pyruvate to lactate *via* lactate dehydrogenase (**3**) increased efflux of the antibiotic (**4**) increased DNA repair capacity (**5**) activation of antioxidant defense systems (**6**) deficiency of the ferrous iron transporter FeoAB, (**7**) overexpression of the rhamnose catabolism regulatory protein RhaR [50].

The exact mechanism of *nim* resistance is not fully understood, and there is conflicting evidence for the role of *nim* genes in metronidazole (MTZ) resistance [50,51]. Figure 4 below shows the involvement of *nim* genes in the mechanism and resistance of metronidazole. The overexpression of *nimA*, *nimE* and *nimJ* induces an increase in MTZ resistance in *E. coli* and *B. fragilis*, respectively [52,53].

However, several *Bacteroides* spp. and genera within the Clostridia class are resistant to MTZ without containing the *nim* gene, while other *nim*-positive species are MTZ-susceptible [54,55,56,57,58]. Thus, the presence of a *nim* gene does not automatically equal resistance. Gal et al. [54] found that only about half of the 50 *Bacteroides* strains positive for the *nim* gene presented minimum inhibitory concentrations (MICs) ranging from 16 to >32 µg/mL—therefore, above the resistance breakpoint. Sethi et al. [59] looked at the MTZ resistance rates in India and the relationship of isolates positive for a *nim* gene with resistance. They found a relatively high rate of resistance (31% compared to <1% in Europe), with a 53% rate of positivity for the *nim* gene. Out of 20 samples, 12 that were positive for the *nim* gene also showed resistance to MTZ, a significant correlation [59]. Leitsch et al. [60] ascertained the levels of expression of the *nim* genes using 2D gel electrophoresis (2DE). The study looked at whether adaptation to an increased concentration of MTZ led to increased levels of the *nim* protein. As *nim* proteins are thought to act as nitroreductases that reduce MTZ to its noncytotoxic form, their profusion should correlate with high levels of MTZ resistance. To test this theory, a high-level MTZ resistance was induced in three strains of *B. fragilis*, but when the *nim* levels were measured, there was found to be no upregulation [60]. Such studies reinforce the complexity of MTZ resistance mechanisms and emphasise that *nim* genes alone are not sufficient to give high-level MTZ resistance [53,54,56]. *Nim* genes have been found in multidrug resistant strains of *B. fragilis*, together with other resistance genes [61]. A cluster of multidrug-resistant (MDR) *B. fragilis* isolates containing *nimB*, *cfiA*, *ermF* and *tetQ* genes was detected using whole-genome sequencing (WGS) [61]. *Nim* genes have also been found in an MDR strain of *B. thetaiotaomicron* together with two *β-lactamase* genes, two *tetX* genes—*tetQ* and *ermF*—two *cat* genes and several genes encoding efflux pumps [62]. Despite this current inability to definitively demonstrate the cause and effect of *nim* genes and MTZ resistance, it is still worthy of further investigation as important risk factors, such as the location of the *nim* genes on mobile genetic materials plus the high usage of MTZ as a frontline drug in multiple infectious diseases could lead to an increase in resistance [50]. This is underlined by the presence of *nim* genes in several MDR anaerobes [63].

## 4. Delamanid and Pretomanid

Delamanid and pretomanid are both bicyclic 4-nitroimidazoles, and phase II clinical trials have shown them to be effective against both replicating and hypoxic nonreplicating mycobacteria [64]. Tuberculosis (TB) is the leading cause of death from infectious disease. In 2018, 10 million people became ill with TB, with 1.2 million deaths [65]. Under aerobic conditions, the mechanism of action is to inhibit the formation of mycolic acids [66], while under anaerobic conditions, the mechanism induces respiratory poisoning [67]. Pretomanid, in particular, donates nitric oxide (NO), which can create toxic conditions within the bacilli in nonreplicating MTB [67]. Both delamanid and pretomanid are prodrugs that require bioactivation of the nitro group to become effective. They are activated in an F420H2-dependant reaction in MTB by deazaflavin-dependant nitroreductase (Ddn) [67,68]. Therefore, mutations that affect the activity of Ddn or the biosynthesis or reduction of F420 may result in resistance [64].

**Figure 5:**
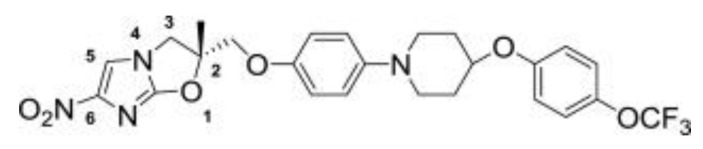
Structure of delamanid [69].

Delamanid (Figure 5) was discovered by Otsuka Pharmaceuticals [69] and was granted conditional approval in 2014 by the European Medicines Agency (EMA) for the treatment of pulmonary MDR-TB in adults in combination with other frontline drugs [70].

The World Health Organisation (WHO) issued a policy guidance, which stated that delamanid could be added to the WHO-recommended regimen [71]. It has a potent minimum inhibitory concentration (MIC) range of 6–240 ng/mL, which is the lowest among the current TB drugs [69,72]; plus, it showed no signs of toxicity in the Ames test [73]. In a phase IIa trial, delamanid showed good activity in drug-susceptible TB patients, but due to poor adsorption at high doses, twice-daily dosing was required (2 × 50-mg tablets per dose) [74,75]. The compound works by inhibiting the synthesis of cell wall components and is active against replicating and nonreplicating persister bacteria, both extra and intracellular bacilli [69]. It does not show a cross-resistance with other TB drugs and can therefore be used in combinations [76]. A phase II trial was completed in 2012 in combination with an optimised background regime (OBR) in 17 centres across nine countries [77]. Phase III trials have since been completed, and further investigations into its use in children are underway [77].

Dormant bacteria are often resistant to treatment, and therefore, new drugs with the ability to kill dormant bacilli are more likely to be effective against MDR-TB. The Wayne model is being used to test the efficacy of TB drugs under low oxygen concentrations [78,79]. Various studies have used a modified version of this model to investigate the bactericidal effects of delamanid in aerobic versus anaerobic settings, with Upton et al. [80,81] showing that it killed 99% of MTB bacilli at 4.4 µg/mL. Delamanid was also as effective as the frontline drug rifampicin against intracellular mycobacteria [82].

**Figure 6:**
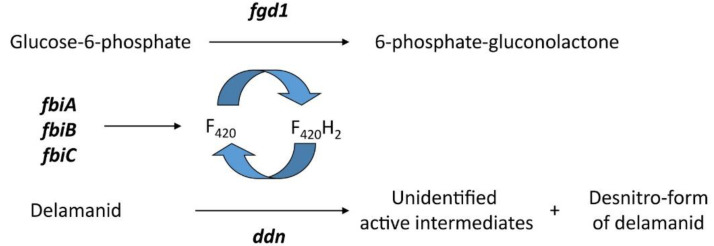
Gene products involved in the bioactivation of delamanid [86].

A resistance to delamanid has been reported in clinical isolates of TB. Bacilli resistant to delamanid have mutations in one of the five genes (Figure 6) associated with the F420-dependant nitroreduction pathway: *fgd1, ddn, fbiA, fbiB* and *fbiC* [83]. *FbiA*, *fbiB* and *fbiC* are coded for proteins FbiA, FbiB and FbiC, which are essential for the biosynthesis of F420, with each gene affecting a different stage [84,85]; *fgd1* (gloucose-6-phospahte dehydrogenase) play a role in the F420 redox recycling mechanism and *ddn* is the reductase required for activation [86].

Fujiwara et al. [86] showed that, from 30 randomly selected resistant colonies, the frequency of mutations across the five genes varied considerably: *ddn* (20%), *fgd1* (30%), *fbiA* (16.7%), *fbiB* (6.7%) and *fbiC* (26.7%). The fact that resistant bacilli lack the ability to activate delamanid is shown by the following; when drug-susceptible MTB bacilli were incubated with delamanid, the concentration decreased as it was converted to the des-nitroimidazole form, but when resistant bacilli were used, no change was seen [82,86].

**Figure 7:**
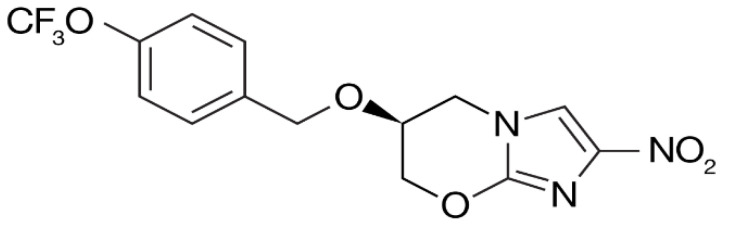
Structure of pretomanid [91].

Pretomanid (Figure 7) was identified by PathoGenesis and was approved by the FDA in 2019 for the treatment of multidrug-resistant TB in combination with bedaquilne and linezolid [87]. The MIC values range from 150 to 250 ng/mL, and no cross-resistance with other TB drugs has yet been seen, making it a good candidate for treating MDR-TB [88]. Studies have shown that pretomanid, in combination with moxifloxacin and pyrazinamide, had better bactericidal activity than the current standard regimen [89,90]. The primary mechanism of action is the inhibition of synthesis of cell wall lipids and proteins [91].

Resistant mutants have shown that both Ddn and FGD1 are essential for the drug to become activated; however, only Ddn is involved in reducing the nitro group, producing three primary metabolites and reactive nitrogen species [67]. A study by Haver et al. [88] looked at 183 spontaneous pretomanid-resistant mutations of which 29% of the lesions were seen in *ddn*, 26% in *fbiC*, 19% in *fbiA*, 7% in *fgd1* and 2% in *fbiB*; the remaining 17% did not show mutations in any of the five genes. Eighty-three percent had single mutations in one of the genes. As 17% had no mutations within the five genes, it can be hypothesised that other targets are involved in either the mechanism of action or activation pathway of pretomanid [88].

As stated previously, mutations that knock out the activity of Ddn completely may result in resistance in both delamanid and pretomanid. However, this could affect the fitness of MTB, as the cofactor F420 has been shown to be essential for the survival of MTB, used by at least 28 enzymes [92] that play important roles in the hypoxic recovery and evasion of the hosts immune system [93,94]. Therefore, in order for nitroimidazole resistance to spread, the native activity of Ddn must either be retained or compensated for. The activation of delamanid and pretomanid by Ddn is a promiscuous activity that is more prone to mutation than native functions [95], meaning that the prodrug activation could be lost without loss of the native function. Lee et al. [96] conducted studies to better understand the fitness costs of the loss of Ddn activity. They were able to demonstrate, through studying 75 mutants, that a number were able to prevent the activation of pretomanid without the loss of all native functions. Therefore, these mutants would be fit enough to spread to new patients [96]. These mutations are likely to be one of the main routes through which resistance to these two new TB drugs will spread. Of the 75 mutants studied, 25 did not reduce pretomanid, but only 10 lost the ability to reduce delamanid, despite their similarities, suggesting that they interact with Ddn in different ways [96]. Clearly, the results from this study should be considered when looking at the continued development and clinical use of nitroimidazoles. Both delamanid and pretomanid are undergoing phase II/III trials, in combination with other drugs, against drug-susceptible TB and MDR-TB. Mutations of Ddn should be closely monitored to ensure that the best drug combination is used, and that will ultimately reduce the spread of resistance. The indication that delamanid binds to Ddn differently than pretomanid means that it could be used to treat some TB strains that are resistant to pretomanid [96]. A combination of the two could also be used to reduce the spread of resistance. Further testing of a range of nitroimidazoles against a variety of Ddn variants could help to identify drugs which are less likely to develop resistance.

To fully understand the resistance mechanism, Fujiwara et al. [86] suggested that a rapid drug susceptibility test should be developed. Presently, only phenotypic tests are available for delamanid based on cultures, with results taking several weeks [97]. However, the development of a molecular based test has been problematic due to the distribution of mutations across five genes [86].

One reason why drugs containing a nitro group have not been more widely used is because of the associations with toxicity, including bone marrow suppression, hepatotoxicity and carcinogenicity [16]. Both types of nitroreduction (I and II) produce toxicity; in type I, this is as a result of the generation of hydroxylamine, and during type II, the generation of reactive oxygen species (ROS) via redox cycling causes pharmacological activity [98]. Therefore, if they are going to play any part in the fight against drug resistance, any new developments must aim to reduce these issues. Drugs such as delamanid and pretomanid have been shown to not be mutagenic or genotoxic, but their use is hampered by issues with solubility. This is an area that could be further investigated by formulation chemists [16].

## 5. Chloramphenicol

The ever-increasing resistance to modern antibiotics has led to the re-evaluation of drugs that had either limited use or were discarded due to toxicity or efficacy issues. One such antimicrobial that is now being reassessed is chloramphenicol [99,100]. Chloramphenicol can cross the blood–brain barrier and other sites that can be hard to target, which makes it a powerful drug in the treatment of bacterial meningitis [99]. However, chloramphenicol has also been linked with haematological toxicity [101], and its use is carefully controlled in developed countries [101]. As a possible consequence of this, it can still be effective against multidrug-resistant organisms, including MRSA [102,103].

The main mechanisms of chloramphenicol resistance occur through functions such as enzymatic inactivation via chloramphenicol acetyltransferance, efflux pump removal and ribosome protection [104]. The bacterial modification of chloramphenicol was first reported only two years after it was discovered. Several species of bacteria were reported as being able to reduce the nitro group, with the resulting compound having no antimicrobial effect [104]. Crofts et al. [104] set out to identify the bacterial genes that could be involved in this reduction mechanism. They chose to focus on type I oxygen-insensitive nitroreductases—in particular, NfsB from *Haemophilus influenzae.* They determined the ability of *H. influenzae* NfsB to reduce the nitro group of chloramphenicol completely to an amine. The reduction is enough to confer resistance to chloramphenicol, but Crofts et al. observed that the *H. influenzae* NfsB enzyme also uses metronidazole (MTZ) as a substrate but does not completely reduce the nitro group. They hypothesised that, while NfsB protects the cell against chloramphenicol, it activates MTZ to its cytotoxic state, therefore offsetting any survival benefits. They concluded that the two drugs could be used together to combat bacteria resistant to chloramphenicol on its own [104].

One of the reasons that chloramphenicol has not been more widely used is the concern that its use could result in the development of aplastic anaemia [105]. The mechanism through which this happens is not fully understood, although it is thought that nitro reduction is involved. The identification of a bacterial enzyme that has been shown to reduce chloramphenicol means that this theory can now be tested and hopefully identify analogues of chloramphenicol that can be used in the clinic [104].

## 6. MT02

The antimicrobial drug MT02 has been identified as a new candidate that is active against Gram-positive bacteria such as *Staphylococcus aureus* [106]. *S. aureus* is a common pathogen associated with a wide range of infections, both superficial and invasive [107]. Together with *S. epidermidis*, it accounts for more than 20% of all infections associated with hospitalisation, affecting over 250,000 patients in the USA and Europe annually [107]. The first antibiotic used to treat *S. aureus* was penicillin; prior to this, the infection was normally fatal [108]. Resistant strains soon appeared, which, in turn, stimulated the search for new antibiotics in the 1950s, such as streptomycin and tetracycline, but resistance soon developed once they were being routinely used in the clinic [109,110]. The development of methicillin was a result of the search for an antibiotic that would be active against penicillin-resistant *S. aureus*. Methicillin-resistant *S. aureus* strains (MRSA) emerged in the 1960s, rendering the drug ineffective [111]. MT02 is a nitro-active compound, and the work carried out by Menzel et al. [106] in 2011 showed that it is a DNA-binding compound leading to the inhibition of DNA replication. It shows high levels of antimicrobial activity against *S. aureus* and other Gram-positive bacteria [106]. El-Hossary et al. [112] carried out further work to identify the potential of *S. aureus* to develop resistance to MT02 [112]. In order to select MT02-resistant clones, *S. aureus* strain MA12 was cultivated with increasing concentrations of MT02. Once the concentration reached 10 µM, a red colour began to appear, which then intensified with increasing concentrations. Work to identify the structure of this red compound showed the stepwise reduction of the aromatic nitro groups of MT02 to amino groups. The conclusion was that this reduction was induced by an enzyme found in the resistant *S. aureus* strain [112]. Four previously identified enzymes with nitroreductase activity (SAUSA300_0788, SAUSA300_0381, SAUSA300_1986 and SAUSA300_2462) were found not to be responsible for this reduction of MT02 [112]. The group carried out total RNA sequencing (RNA-seq), which identified the overexpression of an assumed nitroreductase SAUSA300_0859. This was confirmed by RT-PCR, which showed a 160-fold overexpression of SAUSA300_0859 in the MT02-resistant strain. The importance of SAUSA300_0859 in the development of resistance to MT02 was confirmed when sensitivity was restored by a transposon insertion mutant. A biochemical analysis showed that the SAUSA300_0859-encoded protein produced an intense red colour when incubated with MT02, demonstrating that it is the functional enzyme giving resistance in *S. aureus* [112]. While the molecular mechanism that leads to the overexpression of SAUSA300_0859 is not known, this is thought to be the first example of a nitroreductase-based antibiotic resistance mechanism in *S. aureus*.

## 7. Future Perspectives

As resistances to antimicrobials continue to grow, finding alternatives is crucial. As this review demonstrated, nitroimidazoles have a broad range of activity against many different organisms, and it would therefore be prudent to re-examine their potential. Several strategies that merit further investigation are briefly discussed below.

As well as looking for ways to combat the issue of toxicity, the mechanism of nitroimidazole resistance must be fully understood if effective drugs are to be developed. What is clear from the discussions above is that the role played by nitroreductases needs further investigation. This is of particular importance, as many bacteria have developed resistance to multiple drugs [113]. Strategies that aim to directly combat different resistance mechanisms are more likely to be effective in finding ways to treat drug-resistant organisms. The repurposing of existing antimicrobials and their use in novel combinations is one such strategy. Drug repurposing is defined as “an approved drug in one disease area is found to be active in another disease whereas drug repositioning is the uses of a drug active in one disease as a template for the synthesis of derivatives active in another disease” [114]. Drug rescue is defined as “developing new uses of a drug that failed to progress through clinical studies or which was removed from the market” [114]. All these strategies have been applied to nitroimidazoles.

Collaboration is important in drug development and repurposing. Open databases provide cost-effective access to the sharing of resources and data. Examples include PubChem and Chemspider, which contain chemical structural information, while DrugBank and SuperTarget provide profiles of drugs and target diseases [115]. As NTRs have such a broad spectrum of activity, the use of these databases presents a useful tool. For example, entering “nitroimidazole” as a search in PubChem (December 8th, 2020) identified 3479 molecules in the compound database and 1754 records in the Bioassay database. Research groups can use these databases to profile nitroimidazoles against a much greater number of microorganisms than would have previously been possible. This could potentially identify those with untapped potential. Along similar lines, the open innovation concepts, such as the Community for Open Antimicrobial Drug Discovery (CO-ADD) funded by the Wellcome Trust and The University of Queensland, encourage the open sharing of data and ideas. These initiatives aim to remove any barriers that could limit an individual group’s ability to develop suitable compounds by making resources widely available. This is particularly relevant to NTRs, as their complex modes of action require extensive investigation to fully understand [27].

As previously discussed, NTRs can be used as prodrug activators. Celik et al., [116] investigated the potential for NTRs to be used in antibiotic activation. They aimed to identify possibilities for redesigning existing drugs as precursor prodrugs that could then be activated by bacterial NTRs. They redesigned sulfamethoxazole as a prodrug, and when this was activated by a type I NTR, the results showed an improved antimicrobial action, thus demonstrating that the use of existing approved antibiotics in the form of pre-antibiotics could be used against disease-causing bacteria [116]. The use of antibiotic adjuvants is another strategy that has been shown to work [117]. These are small molecules that, although they have no antimicrobial actions, can increase the efficiency of antibiotics [114].

The development of more robust vaccines for TB would see the need for aggressive treatments with antibiotics reduced. One such area of research is the use of latency-associated antigens, particularly those encoded by the dormancy survival regulator (DosR) regulon [118]. The current BCG vaccine offers poor protection against adult TB [119], as it does not protect against the bacteria when they are in the dormant phase. As most TB patients have a latent infection before any clinical signs are present, latency-associated antigens that are expressed during the dormant phase are of interest as potential vaccine candidates. Kwon et al. [120] selected Rv3131, which is a hypothetical NTR with two DosR-binding sites, as a vaccine candidate. They aimed to investigate whether Rv3131 had any potential as a vaccine against TB. They concluded that it showed promise, as it met the following criteria: it was recognised by the immune system in in vivo experiments, could induce Ag-specific Th-1 T cells and showed activity against highly virulent TB strains [120].

## 8. Conclusions

This review has shown that nitroreductases play an important role in drug activation but are also associated with resistance mechanisms. This review highlighted the urgent need for further investigations to fully understand these mechanisms before they can be utilised against multidrug-resistant organisms for the development of new drugs. This will depend on the committed collaborations between the private and public sectors to translate academic research into the clinic.

## Data Availability

Not applicable.
